# IDH2 is a novel diagnostic and prognostic serum biomarker for non‐small‐cell lung cancer

**DOI:** 10.1002/1878-0261.12182

**Published:** 2018-03-25

**Authors:** Jiang‐jiang Li, Ruilei Li, Wenxiang Wang, Baihua Zhang, Xin Song, Chunfang Zhang, Yang Gao, Qianjin Liao, Ya He, Shuo You, Zheqiong Tan, Xiangjian Luo, Yueshuo Li, Min Tang, Xinxian Weng, Wei Yi, Shifang Peng, Shaohui Liu, Ying Tan, Ann M. Bode, Ya Cao

**Affiliations:** ^1^ Key Laboratory of Carcinogenesis and Invasion Chinese Ministry of Education Xiangya Hospital Central South University Changsha China; ^2^ Cancer Research Institute Xiangya School of Medicine Central South University Changsha China; ^3^ Key Laboratory of Carcinogenesis Chinese Ministry of Health Changsha China; ^4^ Research Center for Technologies of Nucleic Acid‐Based Diagnostics and Therapeutics Hunan Province Changsha China; ^5^ Cancer Biotherapy Center The Third Affiliated Hospital of Kunming Medical University (Tumor Hospital of Yunnan Province) Kunming China; ^6^ The 2nd Department of Thoracic Surgery Hunan Cancer Hospital The Affiliated Cancer Hospital of Xiangya School of Medicine Central South University Changsha China; ^7^ Department of Thoracic Surgery Xiangya Hospital Central South University Changsha China; ^8^ Key Laboratory of Translational Radiation Oncology Hunan Province Hunan Cancer Hospital and The Affiliated Cancer Hospital of Xiangya School of Medicine Central South University Changsha China; ^9^ Second Affiliated Hospital of Xiangya Central South University Changsha China; ^10^ Department of Infectious Disease Xiangya Hospital Central South University Changsha China; ^11^ Health Management Center Xiangya Hospital Central South University Changsha China; ^12^ The Hormel Institute University of Minnesota Austin MN USA; ^13^ National Joint Engineering Research Center for Genetic Diagnostics of Infectious Diseases and Cancer Changsha China

**Keywords:** IDH2, non‐small‐cell lung cancer, serum biomarker

## Abstract

Lung cancer is the most common leading cause of cancer‐related death worldwide. Late diagnosis contributes to a high mortality rate and poor survival of this cancer. In our previous study, we found that IDH2 polymorphism *rs11540478* is a risk factor for lung cancer. Here, we examined IDH2 protein expression in culture medium in which two non‐small‐cell lung cancer (NSCLC) cell lines, H460 and A549, were growing. We found that the IDH2 protein was elevated in the culture supernatant fraction in a time‐ and cell number‐dependent manner. Next, we used ELISA methods to examine IDH2 protein level in serum from patients with NSCLC and healthy controls. We found that IDH2 protein levels in serum could distinguish NSCLC patients from healthy controls with an AUC (area under the curve) of 0.83 (95% confidence interval = 0.79–0.88). The IDH2 level was decreased in serum from NSCLC postsurgical patients compared with the paired presurgical serum. High **s**erum *IDH2* levels appear to correlate with poor survival in patients with NSCLC. These results suggest that IDH2 levels in the serum could be a new effective biomarker for the diagnosis and prognosis of NSCLC.

**Abbreviations**
ELISA: enzyme‐linked immunosorbent assay
IDH2: isocitrate dehydrogenase 2
NSCLC: non‐small‐cell lung cancer

## 1 Introduction

Lung cancer is the leading cause of cancer‐related death worldwide (Jemal *et al*., 2011). Lung cancer has a high mortality rate, which is usually ascribed to late diagnosis (Siegel *et al*., 2012). Late diagnosis also contributes to poor survival of patients with lung cancer (Siegel *et al*., 2012). Age‐standardized one‐year net survival at diagnosis is about 80% in TNM stage I and 20% in TNM stage III non‐small‐cell lung cancer (NSCLC) patients (Walters *et al*., 2013). The five‐year relative survival rates of localized patients with lung and bronchus cancer are about 50%, while for patient with distant metastasis is 4% (Siegel *et al*., 2016). Thus, early diagnosis could greatly reduce mortality rates and increase survival of patients with lung cancer (Ehmann *et al*., 2012).

Isocitrate dehydrogenase 2 (IDH2) is an enzyme that catalyzes the oxidative decarboxylation of isocitrate to α‐ketoglutarate. IDH2 is mainly located in mitochondrial and extracellular exosomes (http://www.genecards.org). Recently, gain‐of‐function mutations of the *isocitrate dehydrogenase 1/2 (IDH)* enzymes have been found in multiple cancers (Clark *et al*., 2016; Flavahan *et al*., 2016; Zou *et al*., 2015). *IDH1/2* mutations could produce the ‘onco‐metabolite’, 2‐hydroxyglutarate (2‐HG) from α‐ketoglutarate (α‐KG) to competitively inhibit α‐KG‐dependent dioxygenases to activate oncogenes and regulate the expression of multiple genes (Ward *et al*., 2010; Xu *et al*., 2011). IDH1/2 inhibitors, such as enasidenib (AG‐221), have been used in more than 11 clinical trials and have shown positive results (Burris *et al*., 2015; Dinardo *et al*., 2015; Fujii *et al*., 2016; Mondesir *et al*., 2016).

Wild‐type IDH1/2 was shown to contribute to cell growth and survival. It could carboxylate α‐ketoglutarate from glutamine in hypoxia and play a role in cell growth and viability, and it was required for lipogenesis in hypoxic melanoma cells (Filipp *et al*., 2012; Wise *et al*., 2011). Glucose and glutamine are the main nutrients utilized for proliferation and NADPH production in cancer cells and can be controlled by oncogenes such as *K‐ras* and *c‐myc* (Cao *et al*., 2011; DeBerardinis *et al*., 2007; Liu *et al*., 2013; Pavlova and Thompson, 2016; Tan *et al*., 2016; Xiao *et al*., 2014; Yu *et al*., 2017). Wild‐type IDH2 participated in reductive carboxylation of glutamine to support redox homeostasis during anchorage‐independent tumor spheroid formation (Jiang *et al*., 2016). In our previous study, we showed that *idh2 rs11540478* is a risk factor for lung cancer(Li *et al*., 2017).

In this study, we first analyzed the protein expression of IDH2 in cell culture medium in which two lung cancer cell lines, NCI‐H460 and A549, were growing and found that the IDH2 level is increased in a time‐ and cell number‐dependent manner. Further, ELISA methods were employed to examine the level of IDH2 in the serum of 296 patients with non‐small‐cell lung cancer and 380 healthy control subjects. We found that the level of IDH2 in serum can differentiate lung cancer patients from healthy controls with an AUC of 0.83 (95% confidence interval = 0.79 to 0.88). The protein level of IDH2 levels was shown to be an indicator of poor survival in patients with NSCLC and decreased after the surgical removal of primary tumors. Overall, these results suggested that serum IDH2 could be a new and reliable biomarker for the diagnosis and prognosis of non‐small‐cell lung cancer.

## 2 Materials and methods

### 2.1 Study design

The study subjects included 200 patients with NSCLC and 380 healthy control subjects who were recruited between 2013 and 2016 from Xiangya Hospital of Central South University and The Third Affiliated Hospital of Kunming Medical University, and 96 patients with NSCLC who were recruited between 2015 and 2017 from Hunan Cancer Hospital. Clinical histopathology confirmed all samples were NSCLC (183 adenocarcinomas and 113 squamous carcinomas) and control subjects were verified to be healthy, based on chest X‐rays, liver function tests, and blood tumor marker analysis. Surgery is the only treatment provided for the patients with NSCLC, 296 serum samples were collected before surgery, and 29 available serum samples were collected 1 week postsurgery. The study protocol was approved by the ethical review committees of the three hospitals. All clinical data including smoking status were obtained from the hospital pathologic records (Table 1).

**Table 1:** Distribution of selected clinical characteristics of NSCLC patients and healthy control subjects

Variable	Lung cancer patients (%) *n* = 296	Control subjects (%) *n* = 380	*P* a
Age (years)			
30–50	50 (16.9)	90 (23.6)	0.09
51–70	205 (69.3)	263 (69.2)	
>70	41 (13.9)	27 (7.1)	
Sex			
Female	133 (44.9)	174 (45.8)	0.79
Male	163 (55.1)	206 (54.2)	
TNM stage			
Ia	200 (67.6)		
Ib	48 (16.2)		
IIa	38 (12.8)		
IIb	10 (3.4)		
Histology type			
Adenocarcinoma	183 (61.8)		
Squamous carcinoma	113 (38.2)		
Smoking status			
Smokers	134 (45.3)	101 (28.9)	0.001
Nonsmokers	162 (54.7)	249 (71.1)	
Unknown		30	

a Two‐sided χ^2^ test.

### 2.2 ELISA

NCI‐H460 (HTB‐177), A549 (CCL‐185), HCT116 (CCL‐247), and HT29 (HTB‐38) were cultured in RPMI 1640 with 10% FBS. Cells were grown in a 37 °C incubator with 5% CO_2_ and seeded into 6‐well plates. Culture medium was centrifuged at 3000 ***g*** for 10 min, and the supernatant was stored at −80 °C.

Serum samples obtained from patients with NSCLC and healthy control subjects were collected using serum separator tubes and centrifuged at 3000 ***g*** for 10 min within 4 h. The supernatant was stored at −80 °C before the final analysis. Serum IDH1/2 assays were performed using a commercially available ELISA kit (SEH839Hu, SEH838Hu 96 Test, Cloud‐Clone) according to the manufacturer's recommendations. The samples and kit components were stored at 4 °C or −20 °C and brought to room temperature before the assay was conducted. Serum samples in standard diluent of a total 100 μL volume were added to the wells of an ELISA analysis plate and covered with a plate sealer and then incubated for 2 h at 37 °C. The liquid was removed, and dilution solution A containing secondary antibodies was added and incubated for 1 h at 37 °C. The plate was washed with buffer 3 times, and then, dilution solution B containing hydrogen peroxide was added and the plate incubated for 30 min. After another five washes with buffer, the substrate 3,3,5,5‐tetramethylbenzidine was added and the plated incubated for an additional 15 min. The stop solution sulfuric acid was added, and the absorbance was immediately measured at 450 nm on a plate reader (BioTek). The concentration of IDH1/2 was calculated using a quadratic polynomial fitting curve.

### 2.3 Statistical analyses

The differences between IDH2 levels in the cell culture medium were analyzed by Student's *t*‐test. The differences between IDH2 levels in serum samples were evaluated by the Mann–Whitney *U*‐test using continuous variables and nonparametric analyses included in GraphPad Prism Windows (version 5). Receiver operating characteristic (ROC) analysis and Cox regression model were performed using SPSS Windows (version 19.0).

## 3 Results

### 3.1 IDH1/2 level in cancer cell culture medium

**Figure 1:**
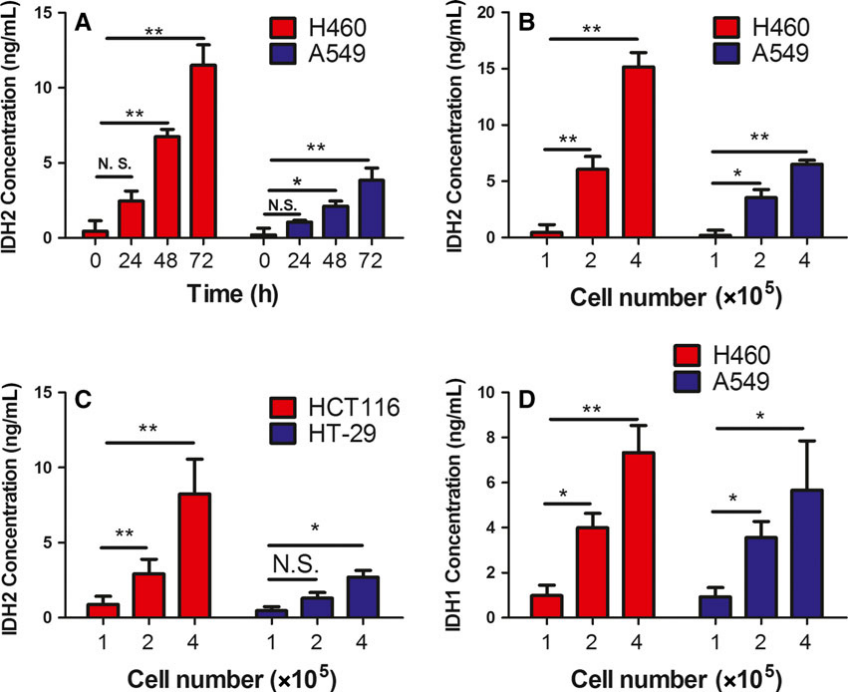
IDH1/2 concentration in the culture medium of cancer cells. The concentration of the IDH2 protein in the culture medium of cancer cells was determined by ELISA methods, and culture medium without cells was used as a control. (A) IDH2 concentration in the culture medium of H460 and A549 lung cancer cells at different culture time points. (B) IDH2 concentration in the culture medium of different numbers of H460 and A549 lung cancer cells. (C) IDH2 concentration in the culture medium of HCT116 and HT29 colon cancer cell lines. (D) IDH1 concentration in the culture medium of H460 and A549 lung cancer cells. The asterisks (*, **) indicate a significant (*P *<* *0.05, *P *<* *0.01, respectively) difference.

IDH2 is mainly located in mitochondrial and extracellular exosomes. The high expression of IDH2 in lung cancer cells and the location of IDH2 suggested that IDH2 might be secreted out of the cells. We analyzed the expression level of the IDH2 protein in cell culture medium in which H460 and A549 NSCLC cells were grown. We found that the levels of IDH2 protein in the culture media from of either cell line increased with culture time (from 24 to 72 h, *P *<* *0.01, Fig. 1A) or with increasing cell number (from 1 × 10^5^ to 4 × 10^5^, *P *<* *0.01, Fig. 1B). The levels of IDH2 protein increased in HCT116 and HT29 cell culture media (*P *<* *0.05, Fig. 1C). The release of IDH2 is not specific to NSCLC, but it could also occur in other cancer cell lines, such as colon cancer cell lines HCT116 and HT29. To determine whether the enzyme secretion/release is isozyme‐specific, we measured IDH1 protein in cultured medium of H460 and A549 cells, which was found to be elevated with cell number (*P *<* *0.01, Fig. 1D).

### 3.2 The level of IDH2 in serum from patients with NSCLC and healthy control subjects

We next recruited a total of 676 participants (including 296 patients with NSCLC and 380 healthy controls, as shown in Table 1). The patients with NSCLC and healthy controls were divided into a training set (96 patients with NSCLC from Hunan province and 130 healthy controls) and a test set (200 patients with NSCLC from Yunnan province and 250 healthy controls).

**Figure 2:**
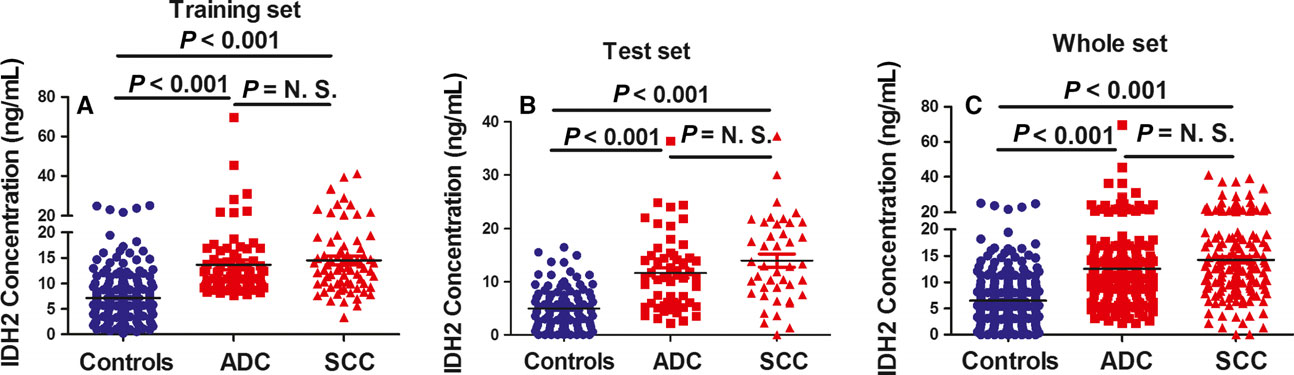
Levels of IDH2 in serum from patients with NSCLC and healthy controls. IDH2 concentration was measured in serum from patients with lung cancer adenocarcinoma (ADC), squamous cell carcinoma (SCC),and healthy controls in training set (A), test set (B), and whole set (C). Statistical significance was determined by the Mann–Whitney *U*‐test.

In the training set, the median level of IDH2 in the serum from patients with NSCLC was 12.50 ±8.16 ng·mL^−1^, which was significantly higher than that of the healthy controls (6.57 ± 5.10 ng·mL^−1^; Fig. 2A; *P *<* *0.0001, Mann–Whitney *U*‐test). The level of IDH2 was also higher in serum from patients with lung adenocarcinoma and squamous cell carcinoma compared with healthy control subjects, but was similar between the two histologic patient groups (Fig. 2A).

In the test set, the median level of IDH2 in the serum from patients with NSCLC was 12.84 ± 7.42 ng·mL^−1^, which was also significantly higher than that of the healthy controls (4.97 ± 3.90 ng·mL^−1^; Fig. 2B; *P *<* *0.0001, Mann–Whitney *U*‐test). The level of IDH2 was also higher in serum from patients with lung adenocarcinoma and squamous cell carcinoma compared with healthy control subjects, but was similar between the two histologic patient groups (Fig. 2B).

In the whole set, the median level of IDH2 in the serum from patients with NSCLC was 12.53 ± 7.76 ng·mL^−1^, which was significantly higher than that of the healthy controls (6.48 ± 4.29 ng·mL^−1^; Fig. 2C; *P *<* *0.0001, Mann–Whitney *U*‐test). The level of IDH2 was also higher in serum from patients with lung adenocarcinoma and squamous cell carcinoma compared with healthy control subjects, but was similar between the two histologic patient groups (Fig. 2C).

Age, gender, and smoking status for both patients and healthy controls were analyzed by Mann–Whitney *U*‐test, and there is no correlation between smoking status and serum IDH2 protein levels both in patients with lung cancer and healthy controls (*P *>* *0.05). There is also no correlation between serum IDH2 protein levels and age, gender, tumor stage (*P *>* *0.05).

### 3.3 ROC analyses of IDH2 in patients with NSCLC and healthy control subjects

Next, ROC curves based on the ELISA results were plotted to assess the potential use of serum IDH2 as a noninvasive biomarker for the diagnosis of patients with NSCLC. Results indicated that the protein level of IDH2 in serum could differentiate the patient with lung cancer from the healthy control subject. We selected an optimum IDH2 cutoff value based on the Youden's J statistic (Youden's index = sensitivity + specificity‐100%) for the diagnosis of patients with NSCLC (Ruopp *et al*., 2008).

**Figure 3:**
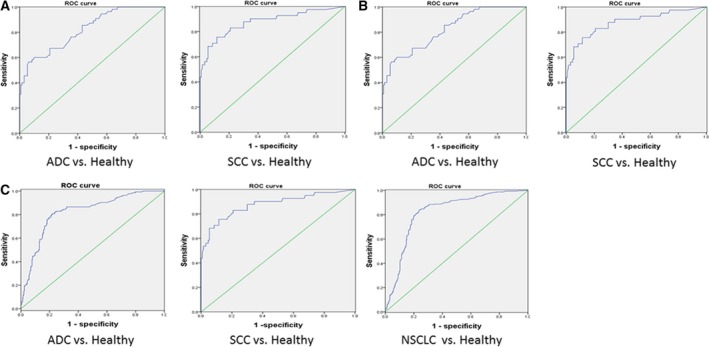
ROC curve analyses using IDH2 to differentiate NSCLC cases from healthy controls. (A–C) ROC curves of patients with lung cancer adenocarcinoma (ADC), squamous cell carcinoma (SCC), and healthy controls in training set (A), test set (B), and patients with NSCLC in whole set (C).

In the training set, the cutoff value was 10.16. The AUC of lung adenocarcinoma was 0.82 (95% CI = 0.79 to 0.88), with a sensitivity of 84.4% and specificity of 78.6% (Fig. 3A). The AUC of lung squamous carcinoma was 0.80 (95% CI = 0.76 to 0.84), with a sensitivity of 80.2% and specificity of 75.2% (Fig. 3A).

In the test set, the cutoff value was 8.34. The AUC of lung adenocarcinoma was 0.82 (95% CI = 0.76 to 0.89), with a sensitivity of 76.6% and specificity of 63.3% (Fig. 3B). The AUC of lung squamous carcinoma was 0.88 (95% CI = 0.81 to 0.95), with a sensitivity of 83.2% and specificity of 79.3% (Fig. 3B).

In the whole set, the cutoff value was 9.51. The AUC of lung adenocarcinoma was 0.82 (95% CI = 0.78 to 0.86), with a sensitivity of 81.5% and specificity of 76.8% (Fig. 3C). The AUC of lung squamous carcinoma was 0.85 (95% CI = 0.81 to 0.89), with a sensitivity of 82.2% and specificity of 77.6% (Fig. 3C). The AUC of patient with NSCLC was 0.83 (95% CI = 0.79 to 0.88), with a sensitivity of 82.1% and specificity of 77.2% (Fig. 3C).

### 3.4 Comparison of IDH2 levels in serum from pre‐ and postsurgical patients with NSCLC

**Figure 4:**
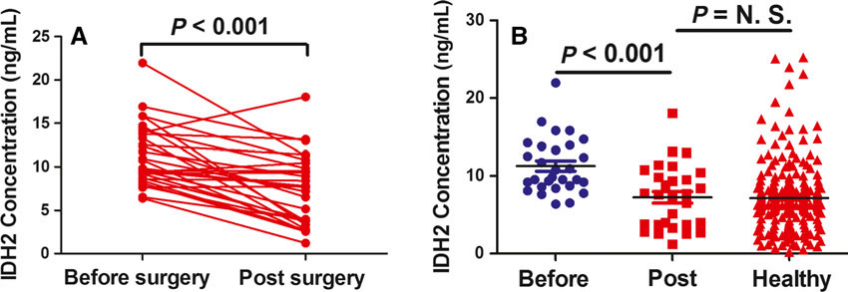
Expression of IDH2 in serum from pre‐ and postsurgical patients with NSCLC. (A) The level of IDH2 in serum was determined by ELISA methods in the same patients with NSCLC before and after surgery (*n* = 29). (B) The level of IDH2 in serum was determined by ELISA methods in healthy control subjects and in the same patients with NSCLC before and after surgery. Statistical significance was determined by the Mann–Whitney *U*‐test.

Surgery is the primary treatment for the TNM stage I and stage II NSCLC patients. We found a level of IDH2 at 7.26 ± 3.98 ng·mL^−1^ in serum from patients with NSCLC at 1 week after surgery, which was significantly lower than level before surgery (11.27 ± 3.51 ng·mL^−1^, *P *<* *0.0001, Mann–Whitney *U*‐test, Fig. 4A) and approached the level of that observed in healthy control subjects (6.57 ± 5.10 ng·mL^−1^, *P *=* *0.91, Mann–Whitney *U*‐test; Fig. 4B).

### 3.5 The association between serum IDH2 levels and overall survival of patients with NSCLC

**Figure 5:**
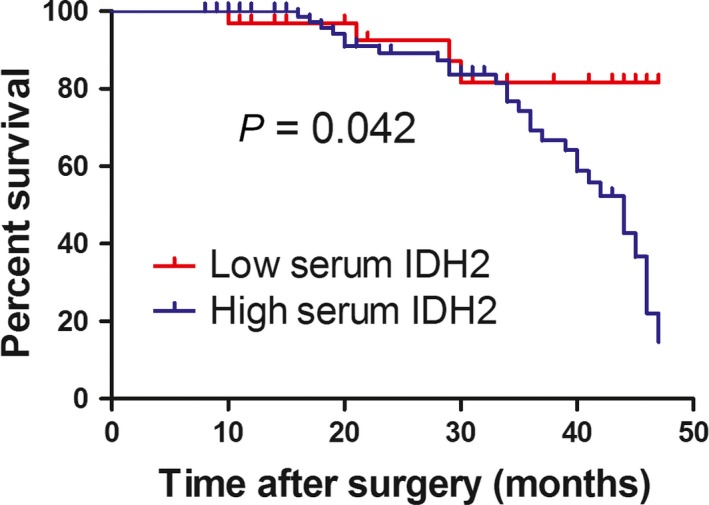
Kaplan–Meier survival analysis of patients with NSCLC based on serum IDH2 levels. The medium of serum IDH2 (12.52 ng·mL^−1^) was set as the cutoff value. The overall survival rate of NSCLC patients with high serum IDH2 level (*n* = 35) was significantly lower than patients with low serum IDH2 level (*n* = 91; *P *=* *0.042; log‐rank test).

Next, we examined the association between serum IDH2 levels with overall survival of patients with NSCLC by performing the Kaplan–Meier survival analysis. We found that patients with higher levels of postsurgical serum IDH2 level had significantly worse overall survival rates (*P *=* *0.042; log‐rank test; Fig. 5). The follow‐up time of these 126 patients was from 10 months to 48 months, with a medium time of 32 months. The Cox regression equation was employed to identify the potential factors that affect overall survival of patient with NSCLC. Male patients were found to survive significantly shorter than female (HR = 0.96, 95% CI = 0.92–0.99, *P *=* *0.025).

## 4 Discussion

Computed tomographic (CT) scans are the most commonly used clinical diagnosis methods for lung cancer, but have some significant limitations, including false‐positive results and over‐diagnosis (Bach *et al*., 2012; Patz *et al*., 2014; Wender *et al*., 2013). Serum protein levels are also used for diagnosing lung cancer. The commonly used clinical serum biomarkers are CEA, CA125, CYFRA 21‐1, SCC, and NSE, but all are not specific (Hanash *et al*., 2011; Molina *et al*., 2003; Niewoehner and Rubins, 2003). Lactate dehydrogenase B in serum was shown to be correlated with the clinical stage of lung cancer (Chen *et al*., 2006). Recently, IDH1 was reported to be up‐regulated in lung cancer tissues and could serve as novel plasma biomarker for the diagnosis of non‐small‐cell lung cancer (Sun *et al*., 2013; Tan *et al*., 2012). None of the published candidate biomarkers for lung cancer diagnosis are yet ready for clinical use (Hassanein *et al*., 2012).

In a previous study, we showed that a new *IDH2* genetic variant *rs11540478* is associated with a risk of lung cancer. Functional analysis showed higher *IDH2* expression and increasing cell viability among T alleles; *IDH2* mRNA was higher in peripheral blood lymphocytes from patients with lung cancer compared to healthy control subjects (Li *et al*., 2017). Here, we found that the IDH2 protein was elevated in a time‐ and cell number‐dependent manner in the culture medium in which lung cancer cells were grown. This suggested that IDH2 might be a protein secreted either in the extracellular exosomes or by other mechanism.

Exosomes can be released by all types of cells, and it is one of the media that cancer cells used to communicate with its neighbor or distant cells, cancer‐associated exosomes can promote tumor growth and invasive (Hoshino *et al*., 2013; Raimondo *et al*., 2015). Due to the function of IDH2 protein, IDH2 in the culture medium may promote proliferation and viability of lung cancer cells. In cell culture process, dying cells may release a part of the extracellular IDH1 and IDH2 in a normal cell turnover process. The dying part of tumor tissues may also release IDH1 and IDH2 protein or spread into blood, which may contribute to increasing of serum IDH2 level and a part of the IDH1 and IDH2 detected.

We found that IDH2 was higher in serum from patients with lung cancer compared to healthy control subjects. Analysis showed an AUC of 0.83 (Fig. 3A), which is better than that of current clinically used biomarkers, including CA125, Cyfra21‐1, and CEA, and similar to that of IDH1 (Sun *et al*., 2013). Recently, serum miRNA and metabolomics have been found to be diagnostic and prognostic biomarker for non‐small‐cell lung cancer. The AUC for miR‐652, miR‐660, miR‐21, and metabolite diacetylspermine (0.74) was 0.82, 0.74, 0.81, and 0.74, respectively, which are nearly similar to that of IDH2 (Wikoff *et al*., 2015; Zhao *et al*., 2015; Zhou *et al*., 2015).

Although the IDH2 levels in patients with NSCLC and healthy controls were significantly different in our study, some overlap was observed between patients and control subjects (Fig. 2). IDH2 can be used as an adjunctive biomarker for diagnosis in lung cancer. Further, the study will also be needed to determine whether serum IDH2 is an accurate biomarker for other cancer types or is specific for NSCLC.

Interestingly, the level of serum IDH2 decreased in patients with NSCLC at about 1 week after surgical removal of the tumor. The level of serum IDH2 was found to be an indicator of poor survival and could serve as a prognostic biomarker for patients with NSCLC. Importantly, it might be served as an effective and accurate biomarker for evaluating the surgical outcome of patients with NSCLC.

## 5 Conclusions

Late diagnosis contributes to a high mortality rate and poor survival of lung cancer. Here, we found IDH2 protein was elevated in the culture supernatant fraction of lung cancer cells. Serum IDH2 protein could be employed to distinguish patients with NSCLC from healthy controls. High IDH2 levels appeared to correlate with poor survival in patients with NSCLC. These results suggested that serum IDH2 could be a new effective biomarker for the diagnosis and prognosis of NSCLC.

## Author contributions

JJL, YH, ZT, XL, YL, MT, XW, and WY performed the experiments. RL, WW, BZ, XS, CZ, YG, QL, SY, SP, SL, and YT recruited the participants. JJL and AMB wrote the manuscript. YC supervised the experiments.
